# Open-label, randomized, non-inferiority clinical trial of artesunate-amodiaquine *versus* artemether-lumefantrine fixed-dose combinations in children and adults with uncomplicated falciparum malaria in Côte d'Ivoire

**DOI:** 10.1186/1475-2875-13-439

**Published:** 2014-11-19

**Authors:** Offianan A Toure, Serge B Assi, Tiacoh L N’Guessan, Gbessi E Adji, Aristide B Ako, Marie J Brou, Marie F Ehouman, Laeticia A Gnamien, M’Lanhoro AA Coulibaly, Baba Coulibaly, Sylvain Beourou, Issiaka Bassinka, Adama Soumahoro, Florence Kadjo, Mea A Tano

**Affiliations:** Malariology Unit, Institut Pasteur de Côte d’Ivoire, PO Box 490, Abidjan 01, Côte d’Ivoire; National Malaria Control Programme, Abidjan, Côte d’Ivoire

**Keywords:** Falciparum malaria AS-AQ, AL, Uncomplicated malaria, Côte d’Ivoire

## Abstract

**Background:**

Emergence of artemisinin resistance has raised concerns that the most potent anti-malarial drug may be under threat. Artesunate-amodiaquine (AS-AQ) and artemether-lumefantrine (AL) are, respectively, the first- and second-line treatments for uncomplicated falciparum malaria in Côte d’Ivoire. Constant monitoring by National Malaria Control Programme (NMCP) of drug efficacy is an important tool in establishing rational anti-malarial drug policies in Côte d’Ivoire.

**Methods:**

In an open label, randomized controlled clinical trial, children and adults were randomized to receive AS-AQ or AL. Both drug regimens were given for three days, and follow-up was for 42 days. The primary endpoint was the 42-day cure rate and was defined as proportion of patients with PCR-corrected cure rate after 42 days of follow-up.

**Results:**

A total of 383 patients who were attending the Anonkoua-koute (Abidjan), Petit Paris (Korhogo) and Libreville (Man) hospitals and presenting with symptomatic acute uncomplicated falciparum malaria were randomized to receive AS-AQ (188) and AL (195). The intention-to-treat analysis showed effectiveness rates of 94.7% and 96.4% for AS-AQ and AL, respectively on day 42. After adjustment for PCR, these rates were 96.8% and 99%, respectively. At day 42, in per-protocol analysis, Adequate clinical and parasitological response (ACPR) PCR uncorrected was 97.8% and 97.4% for AS-AQ and AL, respectively. The PCR adjusted ACPR was 100% for each combination and both regimens were well tolerated.

**Conclusions:**

This study has shown the high efficacy of AS-AQ in patients of all ages with acute uncomplicated falciparum malaria and AS-AQ was non-inferior to AL. Continuous efficacy monitoring is recommended.

## Background

Artemisinin derivatives are advocated for use in anti-malarial combination therapy because they quickly reduce the level of parasitaemia and hence the parasite pool from which resistant *Plasmodium falciparum* strains may arise [1]. Currently, artemether-lumefantrine (AL) and artesunate-amodiaquine (AS-AQ) are the only widely available drugs, which are recommended by most malaria endemic countries in the treatment of uncomplicated falciparum malaria [1]. Prospective periodic monitoring of the efficacy of anti-malarial treatments by *in vivo* studies is part of post-marketing surveillance [2]. Monitoring of artemisinin-based combination therapy (ACT) becomes particularly important in the light of emergence of artemisinin resistance in South-East Asia [3, 4].

In Côte d’Ivoire, AS-AQ and AL have been recommended respectively in first- and second-line treatment since 2005 following earlier studies [5–7]. Continued use of amodiaquine or artesunate monotherapies, persistence of substandard ACT in the private sector [8–10] and resistance to artesunate and/or amodiaquine may jeopardize the future use of AS-AQ and AL as an effective artemisinin-based combination therapy. Constant diligent surveillance with the aim of monitoring the susceptibility of malaria parasites to artemisinin, its derivatives, and partner drugs in endemic areas is, therefore essential. Recently, a molecular marker for the K13-propeller gene has been identified for the monitoring of artemisinin resistance and its derivatives [11]; but effective monitoring still relies largely on *in vivo* studies with adequate follow-up.

The main purpose of this study was to update the baseline data from a randomized clinical trial assessing the safety and efficacy of AS-AQ fixed-dose combination tablets in comparison with a fixed-dose combination of AL, the two drug combinations recommended by the National Malaria Control Programme of Côte d’Ivoire.

## Methods

### Study design

This study was a randomized, open-label, non inferiority clinical trial comparing AS-AQ and AL and was carried out according to current WHO protocol.

### Study sites

This trial was performed from September 2013 through January 2014 at three health care centers in Anonkoua-kouté (southern), Korhogo (northern) and Man (western) in Côte d’Ivoire. *Plasmodium falciparum* transmission is intense and perennial, with recrudescence during the rainy season at every site. *Plasmodium falciparum* is the predominant malaria-causing species.

### Patient screening, recruitment and exclusion

Patients presenting to the site were screened for eligibility and invited to participate in the study if they met each of the following criteria: aged at least six months; body weight ≥5 kg; a history of fever in the previous 24 hours or measured fever (axillary temperature ≥37.5°C or rectal ≥38°C); mono-infection with *P. falciparum*, with parasite density between 2,000–200,000 asexual parasites per microlitre of blood; no other cause of fever than suspected malaria; and no general danger signs or signs of severe and complicated falciparum malaria as per WHO guidelines [12]; able to take study drugs by the oral route; able to attend clinic on stipulated days for follow-up; and signed informed consent (by patient or responsible caregiver).

Exclusion criteria consisted of: presence of severe and complicated malaria as defined by WHO; a mixed plasmodial infection, or concomitant disease masking assessment of the response to anti-malarial treatment; full course of AS-AQ or AL in the past 7 days; and known hypersensitivity to any of the study drugs.

### Baseline evaluation, randomization and treatment

At enrolment, the patient’s symptoms were evaluated. Temperature (axillary or rectal) and weight were measured and a focused physical examination was performed. A thick and thin blood smear for parasitaemia and a blood sample on filter paper (Whatman 3MM) for later molecular analysis were collected before treatment. Patients were randomly assigned to receive either AS-AQ or AL. Both treatments were three-day oral regimens dosed by weight according to the manufacturer’s instructions: AS-AQ 5 to <9 kg: one tablet/day of artesunate (AS) 25 mg/amodiaquine (AQ) 67.5 mg; 9 to <18 kg: one tablet/day of AS 50 mg/AQ 135 mg; 18 to <36 kg: 1 tablet/day of AS 100 mg/AQ 270 mg; ≥36 kg: 2 tablets/day of AS 100 mg/AQ 270 mg.

AL tablet strength was 20 mg artemether/120 mg lumefantrine: 5 to <15 kg: 1 tablet/dose; 15 to <25 kg: 2 tablets/dose; 25 to <35 kg: 3 tablets/dose; ≥35 kg 4 tablets/dose. AL was administrated twice a day.

The first dose was taken at enrolment, the second dose eight hours later on day 0, and then two doses at 12-hourly intervals for the subsequent two days. All tablets were either swallowed whole or crushed with water. Yoghurt was then given with AL to the participants. The drug administration was supervised by the research team. Randomization was done by an independent statistician using computer-allocated blocks of ten and was not stratified. Individual treatment allocations were contained inside consecutively numbered sealed envelopes, which were opened sequentially by a study investigator or clinical research coordinator after the decision to enroll a subject had been made by the study team. In the case where a participant vomited the first dose within 30 minutes of drug administration, a repeat full dose was re-administered. If vomiting persisted, the patient was withdrawn from the study. A participant who was withdrawn due to vomiting received rescue medication according to the Malaria Control Programme guidelines.

### Sample size

Based on previous data for AL efficacy [13, 14], efficacy rate, defined as the PCR-adjusted parasitological cure rate at day 42, was estimated of 95% for both treatments. The maximal difference acceptable for the AS-AQ to be considered as clinically non-inferior is 10% (absolute value in percentage). For a statistical power of 90% (β =10%), a risk α =5% and using 95% Confidence Interval, sample size for each arm was 162 per treatment group. Allowing for 10% incidence of premature withdrawals, the total number of patients required would be 178 per treatment group.

### Laboratory evaluations

Thick and thin blood smears were stained with 10% Giemsa for 30 minutes. Parasite density was determined by reading the thick blood smear and counting the number of asexual parasites and the number of leukocytes for 200 high-powered fields. Slides were considered negative if no parasite was detected after reading 200 high-powered fields. Presence of gametocytes was also recorded. Thin blood smears were reviewed for non-falciparum infections. Two microscopists independently read all slides and parasite densities were calculated by averaging the two counts.

Readings with discordant results (difference in species diagnosis, difference in parasite density of 25%, or any difference that affected recruitment or study outcome) were re-examined by a third microscopist; the parasite density was calculated by averaging the two closest densities while the final parasite species was determined by the two concordant reads.

Blood samples were drawn for haematology (haemoglobin level, leucocyte count, haematocrit) and biochemistry (serum alanine aminotransferase, aspartate aminotransferase, total bilirubin, glycaemia, urea and creatinine) assessments on day 0, 3 and on the day of recurrent parasitaemia. Genotyping was done according to international recommendations [15].

Blood spots on Whatman 3 M filter paper were prepared for PCR genotyping. Multiplicity of infection was assessed using three polymorphic loci (merozoite surface proteins 1 and 2 and glutamate-rich protein). Briefly, filter paper blood samples collected on the day of enrolment and on the day of failure were analysed for polymorphism of merozoite surface protein-1 (*msp-1*) and merozoite surface protein-2 (*msp-2*) using nested-PCR as previously described [15]. Possible outcomes were new infection or recrudescence. A “new infection” is a subsequent occurring parasitaemia in which all the alleles in parasites from the post-treatment sample are different from those in the admission sample, for one or more loci tested. In a “recrudescence,” at least one allele at each locus should be in common for both paired samples.

When no amplification was observed for either of the paired samples, the outcome was classified as indeterminate.

### Follow-up procedures and classification of treatment outcomes

The patients returned on days 1 and 2 to complete the drug administration and for clinical assessment. They were also given appointment for days 3, 7, 14, 21, 28, 35 and 42 for clinical examination, blood smears and filter paper confection. Patients who failed to report at the clinic for the scheduled visit were followed to their residence by trial field workers. They were also asked to return to the clinic on any other day if new complaints, or any change in their condition.

Treatment outcomes were classified according to the WHO guidelines for areas of intense transmission as adequate clinical and parasitological response (ACPR), early treatment failure (ETF), late clinical failure (LCF) and late parasitological failure (LPF) [2]. Failure was defined as the sum of ETF, LCF and LPF. The primary endpoint was the 42-day cure rate and was defined as proportion of patients with PCR-corrected cure rate after 42 days of follow-up. Secondary efficacy endpoints included, fever and parasite clearance time, gametocytaemia (prevalence and density) at day 7, 14, 21 and 28.

### Safety assessments

Safety outcomes included risk of adverse events. At each follow- up visit, any new or worsening event and laboratory results were assessed passively and actively. An adverse event was defined as any unfavorable and unintended sign (including an abnormal laboratory finding), symptom, or disease temporarily associated with the use of an investigational product, not present on day 0, but occurred during follow-up, or was present on day 0 but became worse during follow-up. Serious adverse event was defined as any event that resulted in patient hospitalization, death, life threatening experience, persistent/significant disability, or specific medical/surgical intervention to prevent serious outcome.

### Statistical analysis

Data generated were recorded in a log book and individual participants case record files. Data were entered and analysed with EPI-Info version 6.4. An intention to treat (ITT) and per protocol (PP) analysis were done. The ITT population included all randomized subjects who received any study medication and was the same as the safety population. Patients with major violations and patients lost during the follow-up or withdrawn (due to an adverse event or to the use of another drug with anti-malarial activity or withdrawal consent) have been considered as failure. The PP population included patients that received a full course of study medication, had a known day-42 primary endpoint, and did not violate the protocol so as to impair evaluation of the primary endpoint. Those lost to follow up, and the withdrawals of consent were excluded from the per protocol analysis. Frequencies were compared by either chi-squared or Fisher’s exact tests, as appropriate, and continuous variables by Student’s *t*-tests when the data are normally distributed. No normally distributed data were transformed to normality.

### Ethical issues

The study was conducted in accordance with the local laws and regulations, International Conference on Harmonization - Good Clinical Practice (ICH-GCP). The protocol was reviewed and approved by the Comité National d’Ethique et de Recherche de Côte d’Ivoire (N°56/MSLS/CNER-dkn). Written informed consent was obtained from participants/parents/guardians. In case of an illiterate patient, his/her thumb impression and signature of an independent witness were obtained.

## Results

### Study trial profile and baseline characteristics

**Figure 1:**
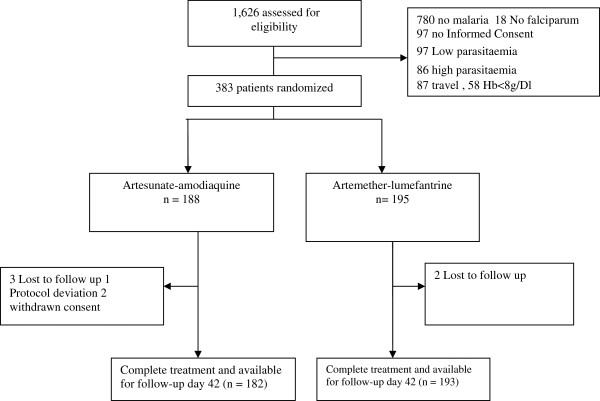
**Trial profile.**

The trial profile is summarized in Figure 1. A total of 1,626 patients were screened of whom 383 fulfilled the inclusion criteria: 188 in the AS-AQ group and 195 in the AL group. Three Lost to follow up, one Protocol deviation and two cases of withdrawn consent were registered in the AS-AQ group when only two lost to follow up were noted in AL group. A total of 182 patients ended up in AS-AQ group and 193 patients in AL group.

The baseline characteristics in the ITT cohort of both groups were similar (Table 1) regarding age, gender, weight, axillary or rectal temperature and parasite density. The average age was 15.7 ± 16.2 in AS-AQ group and 14.4 ± 12.1 in AL group (p = 0.36). Average weight was 36.1 ± 23.3 in AS-AQ group and 36.6 ± 21.8 in AL group (p = 0.80). The sex ratio was not significantly different: 1 in AS-AQ group and 0.7 in AL group (p = 0.09). Overall, both groups of patients were comparable at the inclusion as far as sociodemographic parameters are concerned.

**Table 1:** **Baseline characteristics in the ITT cohort**

	AS-AQ		AL		***p value***
Number of patients	188		195		-
Mean age (SD)	15.7	(±16.2)	14.4	(±12.1)	0.36^a^
Sex ratio (M/F),	1.04		1.35		0.09^b^
Mean weight, kg (SD)	36.1	(±23.3)	36.6	(±21.8)	0.80^b^
Mean temperature, °C (SD)	38.3	(±0.81)	38.3	(±0.9)	0.74^b^
GM parasite count, μL (SD)	38,752.8	(±47,806.9)	41,371	(±65,165.8)	0.65^b^

Notes: ^a^chi-squared test; ^b^Student’s *t*-test.

Abbreviations: AS-AQ, artesunate-amodiaquine; AL, artemether-lumefantrine; GM, geometric mean, ITT, intention to treat.

### Fever and parasite clearance

**Figure 2:**
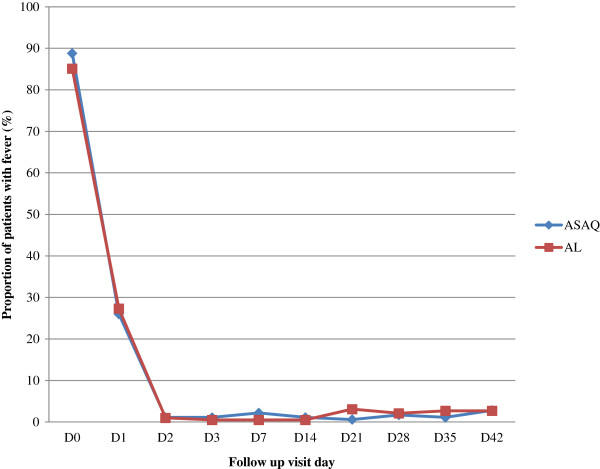
**Proportion of patients with fever during follow up.** Note: No significant differences were found for the proportions of subjects with fever between the two treatment regimens from baseline to day 28 (*P* >0.05 Fisher’s exact test or chi-squared test as appropriate). Abbreviations: AL, artemether-lumefantrine; AS-AQ, artesunate-amodiaquine.

**Figure 3:**
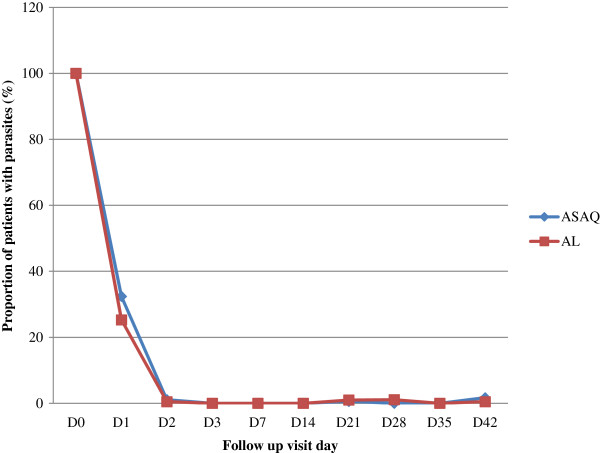
**Proportion of patients with parasites during follow up.** Note: The proportion of participants who were aparasitaemic was similar between the two treatment arms: 98.9% and 99.5% (*P* =0.49) for those receiving AS-AQ or AL, respectively, on day 2. On day 3 these rates were 100% in the two arms. Abbreviations: AL, artemether-lumefantrine; AS-AQ, artesunate + amodiaquine.

As shown in Figure 2, the proportion of participants without fever was similar on days 2 and 3. On day 2, the fever clearance rates were 98.9% (180 of 182) and 99% (191 of 193) in the AS-AQ and AL groups, respectively (p >0. 5), while on day 3, these rates were 98.9% (180 of 182) and 99.5% (192 of 193). Both treatments resulted in rapid clearance of parasites (Figure 3) as parasite clearance rates on day 2 were 98.9% (180 of 182) and 99.5% (192 of 193) in the AS-AQ and AL groups, respectively. At day 3 parasite clearance rate was 100% in both groups.

### Gametocyte carriage

**Figure 4:**
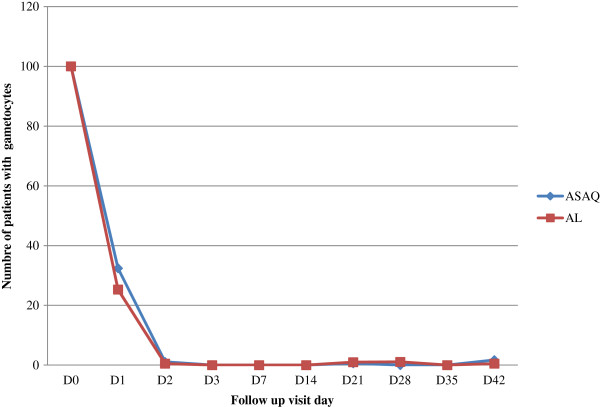
**Proportion of patients with gametocytes during follow-up. Abbreviations: AL, artemether-lumefantrine; AS-AQ, artesunate-amodiaquine.**

At enrolment, gametocytes were detected in the peripheral blood of 10 (5.3%) and 9 (4.6%) patients in the AS-AQ and the AL group respectively. The gametocyte carriage rate was 3.3% (six patients) and 2.1% (four patients) on day 3. All patients were free of gametocytes from day 28 to day 42 in the AS-AQ group and from day 14 to day 42 in the AL group (Figure 4).

Five patients did not have gametocytes at enrolment developed gametocytaemia after treatment: two patients in AS-AQ group and one AL group at day 1, at day 2 Two patients in AS-AQ group and one patient at day 7 in AL group.

### Therapeutic efficacy

No early treatment failure (ETF) was observed in either study group. Adequate clinical and parasitological responses were similarly high in both treatment groups. Until day 42, adequate clinical and parasitological response was observed in patients: 97.8% (178/182) in the ASAQ group and 97.4% (188/193) in the AL group. Nine of three hundred seventy five patients who completed at least 42 days follow-up had reappearance of parasitaemia (five in the AL group, four AS-AQ group) after initial clearance of parasitaemia between days 21 and 42. Among the nine patients with recurrent parasitaemia after day 21, PCR genotyping was successfully performed on all blood samples.

The ACRP at day 42 after PCR correction in ITT analysis was 96.8% and 99% for AS-AQ and AL, respectively. In PP analysis, this rate was 100% for both drugs. Thus, the non-inferiority of AS-AQ was shown both in ITT and PP analysis (Table 2). Regarding treatment failure, after PCR correction, 2.2% (4 patients) and 2.6% (5 patients) in AS-AQ group and in AL group, respectively, were considered as reinfections. Any difference was obtained in the therapeutic efficacy analysis done per site (Table 3).

**Table 2:** **Treatment outcome of AS-AQ** ***versus*** **AL at D42**

Outcome	AS-AQ	AL	***p value***
**ITT Analysis**			
Enrolled patients	188	195	
Patients seen at day 42	182/188 (96.8%)	193/195 (99%)	0.13
Missing	06/188 (3.2%)	02/195 (1%)	0.13
Crude failure rate at day 42	04/188 (2.1%)	05/195 (2.6%)	0.77
Crude cure rate at day 42	178/188 (94.7%)	188/195 (96.4%)	0.41
PCR adjusted failure rate at day 42	06/188 (3.2%)	02/195 (1%)	1
PCR adjusted cure rate at day 42	182/188 (96.8%)	193/195 (99%)	0.32
**PP analysis**			
Patients seen at day 42	182	193	
Crude failure rate at day 42	04/182 (02.2%)	05/193 (2.6%)	0.80
Crude cure rate at day 42	178/182 (97.8%)	188/193 (97.4%)	0.80
PCR adjusted failure rate at day 42	0/182 (0%)	0/193 (0%)	1
PCR adjusted cure rate at day 42	182/182 (100%)	193/193 (100%)	0.59

**Table 3:** **Treatment outcome stratified by site**

Treatment outcome stratified by locality	Artesunate/Amodiaquine	Artemether/Lumefantrine	p value
**ABIDJAN (130 patients)**	**65**	**65**	
**ITT analysis**			
Crude failure rate at day 42	3/65 (4.61%)	00/65 (00%)	0.07
Crude cure rate at day 42	62/65 (95.4%)	65/65 (00%)	0.07
PCR adjusted failure rate at day 42	1/65 (1.5%)	00/65 (00%)	0.31
PCR adjusted ACPR at day 42	64/65 (98.5%)	65/65 (100%)	0,31
% difference	1,5 [-1 ; 4]		
**PP analysis**			
Crude failure rate at day 42	2/64 (3.1%)	00/65 (00%)	0.31
Crude cure rate at day 42	62/64 (96.9%)	65/65 (00%)	0,31
PCR adjusted failure rate at day 42	00/64 (00%)	00/65 (00%)	1
PCR adjusted ACPR at day 42	64/64 (100%)	65/65 (100%)	1
% difference	0		
**KORHOGO (128 patients)**	**64**	**64**	
**ITT analysis**			
Crude failure rate at day 42	03/64 (4.7%)	03/64 (4.7%)	1
Crude cure rate at day 42	61/64 (95.3%)	61/64 (95.3%)	1
PCR adjusted failure rate at day 42	00/64 (00%)	00/64 (00%)	1
PCR adjusted ACPR at day 42	61/64 (95.3%)	64/64 (100%)	0.07
% difference	4,7[0 ; 9]		
**PP analysis**			
Crude failure rate at day 42	00/61 (00%)	03/64 (4.7%)	0.07
Crude cure rate at day 42	61/61 (100%)	61/64 (95.3%)	0.07
PCR adjusted failure rate at day 42	00/61 (00%)	00/64 (00%)	1
PCR adjusted ACPR at day 42	61/61 (100%)	64/64 (100%)	1
% difference	0		
**MAN (125 patients)**	**59**	**66**	
**ITT analysis**			
Crude failure rate at day 42	02/59 (3.4%)	4/66 (6%)	0.47
Crude cure rate at day 42	55/59 (93.2%)	62/66 (94%)	0.47
PCR adjusted failure rate at day 42	02/59 (3.4%)	02/66 (3%)	0.90
PCR adjusted ACPR at day 42	57/59 (96.6%)	64/66 (97%)	0,90
% difference	0,4[-5 ; 6]		
**PP analysis**			
Crude failure rate at day 42	02/57 (3.5%)	02/64 (3.1%)	0.90
Crude cure rate at day 42	55/57 (96.5%)	62/64 (96.9%)	0.90
PCR adjusted failure rate at day 42	00/57 (00%)	00/64 (00%)	1
PCR adjusted ACPR at day 42	57/57 (100%)	64/64 (100%)	1
% difference	0		

### Tolerability and safety

Overall, AS-AQ and AL were well tolerated in this study. Most of the recorded adverse events were signs and symptoms of malaria, thereby making their assessment difficult. None of the patients presented with serious adverse events that necessitated withdrawal from the study or hospitalization was observed in both groups. Adverse effects associated with the gastrointestinal tract, such as anorexia (21.3% *vs* 10.3%), Nausea (3.2% *vs* 0%) vomiting (8.5% *vs* 1%), diarrhoea (5.8 %,) and abdominal pain (13.8% *vs* 6.7%) were more frequent in the AS-AQ group compared to the AL group (p < 0.05). Others clinical adverse events were asthenia (50.5% *vs* 22.3%) and dizziness (7.4% *vs* 1%) and were more frequent in AS-AQ group (p < 0.05). All of these events were described as mild or moderate. Table 4 shows details of the distribution of clinical AEs recorded during the study.

**Table 4:** **Clinical adverse events in the ITT population**

Adverse events	AS-AQ			AL			Fisher’s Exact Test
	n	%	95% IC	n	%	95% IC	***p value***
Asthenia	95	50.5	41.72-59.33	43	22.1	14.75-29.34	<0.0001
Headache	18	9.6	04.39-17.70	22	11.3	05.71-16.84	0.58
Anorexia	40	21.3	14.06-28.47	20	10.3	04.91-15.58	0.003
Abdominal pain	26	13.8	07.74-19.89	13	6.7	02.27-11.04	0.02
Nausea	6	3.2	0.09-06.28	0	0	-	0.01
Vomiting	16	8.5	03.59-13.42	2	1	-	0.001
Drowsiness	5	2.7	-	1	0.5	-	0.09
Dizziness	14	7.4	02.82-12.05	2	1	-	0.002
Rash	0	0	-	1	0.5	-	0.32

Notes: The most commonly reported and possibly drug-related adverse events to both combination therapies were gastrointestinal effects (abdominal pain, anorexia, nausea, abdominal pain, and vomiting); *P* values were obtained by Fisher’s exact test.

Abbreviations: AL, artemether-lumefantrine; AS-AQ, artesunate-amodiaquine.

Frequent laboratory AEs included mild or moderate hyperbilirubenia, hypercreatinemia or anaemia in both arms at day 3 was observed (Table 4). The prevalence of anaemia at baseline and day 3 was, respectively, 58.6% and 78.9.3% in the AS-AQ group. In the AL group this prevalence was 57.4% and 79.5% at day 0 and day 3, respectively. Liver abnormalities were almost exclusively asymptomatic and mildly increased AST and/or ALT values. More participants presented increase of AST and/or ALT at day 3 compared to day 0 in both arms (Table 5).

**Table 5:** **Biological parameters evolution between day 0 and day 3**

	AS + AQ			AL		
Parameters	D0	D3	***P value***	D0	D3	***p value***
Mean Hb level (g/dL) (±SD)	10.53 (±1.98)	9.39 (±2.74)	<10^-4^	10.57 (±1.84)	8.72 (±2.88)	<10^-4^
Anaemia (Hb level <11 g/dL) (%)	58.6	70.9	0.014	57.4	79.5	<10^-4^
Patient with abnormal ALAT (<40UI/L) (%)	79.79	90.40	0.012	75.90	81.89	0.20
Patient with abnormal ASAT (<40UI/L) (%)	55.32	84.07	<10^-4^	51.79	79.47	<10^-4^
Mean bilirubin level (±SD)	06.45 (±6.65)	08.26 (±8.30)	0.14	5.50 (±4.81)	8.89 (±9.93)	0.01
Mean creatinine level (±SD)	9.50 (±5.75)	16.03 (±17.64)	<10^-4^	8.70 (±2.51)	13.68 (±10.86)	<10^-4^

Abbreviations: AS-AQ, artesunate-amodiaquine; AL, artemether-lumefantrine; D, day; Hb, hemoglobin; ASAT, aspartate amino transferase; ALA, alanine amino transferase.

## Discussion

Introduction of highly efficacious artemisinin-based combination therapy (ACT) as first-line treatment in most malaria endemic countries has contributed to recent notable reversals of trends in childhood morbidity and mortality [1, 16]. Because of the prominent value of ACT in current malaria control programmes, the emergence of parasite resistance to artemisinins and the associated compromised efficacy of ACT would pose a major public health problem. The recently reported emergence of artemisinin-resistant malaria characterized by slow initial parasite clearance and high rates of recrudescent infections in Western Cambodia and other countries in South East Asia is, therefore, of great concern [3, 17, 18].

The present study was conducted to provide supporting evidence for the clinical efficacy of AS-AQ and AL, which were adopted and implemented as anti-malarial drug policy in Côte d’Ivoire since 2005. These two forms of ACT have been frequently prescribed in health services and widely available and taken for self-medication since this date. Current international guidelines advocated the need to monitor ACT regularly in order to detect early signs of declining efficacy, which can have implications for policy markers [2].

The study was based on the *in vivo* efficacy using 42-follow up days protocol of two different forms of ACT, AS-AQ and AL among children and adults with uncomplicated falciparum malaria in NMCP three sentinels sites (Abidjan in the south, Korhogo in the north and Man in the west) in Côte d’Ivoire. The day 42 genotyping-adjusted cure rate estimates of AS-AQ and AL reached 100% for each drug. Both treatments were well above the WHO recommended 90% threshold for treatments in uses [2].

These results are in agreement with earlier studies in Côte d’Ivoire that have shown for both combinations high cure rate [6, 19–21] and are also consistent with the high cure rates that have been reported for ACT in other malaria-endemic areas of Africa [19, 22–25]. In addition to high cure rates, most of the other parameters for assessing efficacy such as PCT and FCT were also comparable between the two groups.

The proportion of parasitaemic patients on day 3 has been reported as an interesting indicator for monitoring artemisinin resistance [26]. Although this indicator requires large sample sizes and accurate timing of sample collection for precise estimates, PCT for both AS-AQ and AL in the present study, was nevertheless reassuring. It is, however, noteworthy that the FCT and PCT in the present study were inaccurate measures because body temperature and parasite density were measured every 24 h from day 0 to day 3 and not every 6 or 8 h as in some other published studies [27]. The use of more accurate estimations of parasite clearance rates with recently developed tools should be preferred but demand repeated parasitaemia measures [28].

Mild asthenia AEs were very frequently reported in both treatment arms, with almost twice as many patients in the AS + AQ arm than in the AL arm (50.5% vs 22.3%), p <0.05). Asthenia is considered among common undesirable effects of AS-AQ [29]. No differences in fatigue or asthenia between AS-AQ and AL in some studies [5, 25] were observed. AS-AQ presents the advantage of requiring one intake per day without fatty food while AL is twice per day with fatty food. However, the tolerability, in particular asthenia and gastro-intestinal disorders may impact the effectiveness of the treatment and should be assessed, particularly while used in home management of malaria [30] or as intermittent preventive therapy in children [31].

Regarding biological aspects, a significant increase in the number of patients presenting an Anaemia, abnormal ASAT and ALAT has been observed in both groups. These abnormalities have been also observed in other studies with AS-AQ and AL [20, 23].

Safety and tolerability monitoring of AS-AQ, AL and other forms of ACT should continue in a standardized manner. Pharmacovigilance networks are not implemented in most settings where ACT is routinely used. There are some limitations concerning this study. Drug levels were not tested and only first doses of AL were observed. As absorption is fat dependent, fatty food was provided and patients were precisely instructed how and when to take the rest of the medication. However, intake was not directly controlled.

## Conclusion

AS-AQ and AL were safe and effective drugs for the treatment of uncomplicated falciparum malaria in the study areas nine years after the deployment of these drugs. AS-AQ and AL showed rapid fever and parasite clearance and the efficacy met the WHO criteria for efficacy (*>*90%) in malaria endemic regions. The efficacy of these two different forms of ACT needs to be carefully monitored periodically since the treatment failures can occur due to resistance as well as sub-therapeutic levels due to inadequate absorption or low adherence to the drug.

## Abbreviations

ETF: Early treatment failure
ACPR: Adequate clinical and parasitological response
ACT: Artemisinin-based combination therapy
LCF: Late clinical failure
LPF: Late parasitological failure
NMCP: National Malaria Control Programme
WHO: World Health Organization
AS-AQ: Artesunate-amodiaquine. AL, Artemether-lumefantrine
FCT: Fever clearance time, PCT, Parasite clearance time.
